# Clinical and epidemiological risk factors for ventilator-associated pneumonia in a cohort of critically ill patients

**DOI:** 10.1186/cc10680

**Published:** 2012-03-20

**Authors:** G De Pascale, MA Pennisi, V Raggi, E Piervincenzi, V Bernini, A Occhionero, P De Santis, A Moccaldo, S Cicconi, R Maviglia, M Tumbarello, M Antonelli

**Affiliations:** 1 Sacro Cuore Catholic University, Rome, Italy

## Introduction

Ventilator-associated pneumonia (VAP) represents a major infectious complication in the ICU. The aim of this study is to identify risk factors for VAP acquisition.

## Methods

All patients admitted to the 18-bed ICU of our university hospital between 1 October 2009 and 31 December 2010 were enrolled on the day of VAP diagnosis. Controls were selected by our computerized database. Statistical analyses were performed using the StataICl l program.

## Results

Over the study period, among 902 admissions, 100 VAP occurred. The rate of multidrug resistance (MDR) was 23%. Development of VAP was associated with a significantly longer duration of ICU stay (24 days (17 to 30) vs. 7 days (5 to 9); *P *< 0.001) and mechanical ventilation (19 days (13 to 20) vs. 4 days (3 to 6); *P *< 0.001). Overall ICU mortality was higher in the VAP population (41% vs. 29%; *P *= 0.09). Comparing patients affected by VAP with controls (100 matched patients), the former group was significantly more likely to be male (*P *< 0.001) and to be immunosuppressed (*P *= 0.004). In addition, VAP development was associated with higher rate of central venous catheter placements (*P *< 0.001), higher mean SOFA score value (*P *< 0.001) and previous exposure to antimicrobials (*P *= 0.004). Successful use of noninvasive ventilation, and trauma admission appeared as protective factors (*P *< 0.001). Table 1 shows independent risk factors associated with VAP acquisition in multivariate analysis.

**Table 1:** 

	*P *value	OR (95% CI)
NIV success	0.005	0.1(0.01 to 0.4)
SOFA score*	0.01	1.2(1 to 1.3)
Male gender	<0.001	14(5 to 39.4)
Immunosuppressive status	0.001	4(1.7 to 9.6)

*Mean value.

## Conclusion

VAP occurrence seems to be associated with increased morbidity and ICU mortality. NIV use, avoiding endotracheal intubation and invasive mechanical ventilation, has appeared to be effective in reducing the rate of VAP episodes, particularly in high-risk patients (severe immunosuppressed). The application of behavioural intervention bundles might represent the suitable preventive measure in settings where high rates of MDR pathogens limit the extensive use of pharmacological ones.
